# A Study of the Correlation between VEP and Clinical Severity in Children with Autism Spectrum Disorder

**DOI:** 10.1155/2018/5093016

**Published:** 2018-01-14

**Authors:** Winai Sayorwan, Nutthida Phianchana, Kannika Permpoonputtana, Vorasith Siripornpanich

**Affiliations:** ^1^Kanchanabhishek Institute of Medical and Public Health Technology, Nonthaburi 11150, Thailand; ^2^Research Center for Neuroscience, Institute of Molecular Biosciences, Mahidol University, Nakhon Pathom 73170, Thailand; ^3^Department of Occupational Therapy, Faculty of Physical Therapy, Mahidol University, Nakhon Pathom 73170, Thailand

## Abstract

Visual evoked potential (VEP) is a technique used to assess the brain's electrical response to visual stimuli. The aims of this study were to examine neural transmission within the visual pathway through VEP testing in preschool children with autism spectrum disorder (ASD) and compare it to age-matched controls, as well as search for a correlation between the VEP parameters and the symptoms of ASD. Participants were composed of ASD children (9 males) and typically developing children (8 males and 4 females), aged between 3 and 5 years. Checkerboards were chosen as the pattern-reversal VEP. The clinical severity of ASD was assessed using the Autism Treatment Evaluation Checklist (ATEC) and the Vineland Adaptive Behavior Scales 2nd edition (VABS-II). Our findings demonstrated that children with ASD had significantly longer N145 latency compared to the controls. A longer N145 latency correlated with a higher score of ATEC within the sensory/cognitive awareness subdomain. In addition, a slower N145 response was also associated with a lower VABS-II score within the socialization domain. The correlation between longer VEP latency and abnormal behaviors in children with ASD suggests a delayed neural communication within other neural circuits, apart from the visual pathway. These lines of evidence support the possibility of using VEP, along with clinical parameters, for the assessment of ASD severity.

## 1. Introduction

Autism is a common neurodevelopmental disorder characterized by impaired communication, deficits in social skills, and abnormal behaviors. The current version of the Diagnostic and Statistical Manual of Mental Disorders (DSM) by the American Psychiatric Association (APA), the DSM-5, collapses various diseases within the group of pervasive developmental disorders (PDD), including autism disorder, into a single disorder, the autism spectrum disorder (ASD) [1]. The diagnosis of ASD is strongly dependent on clinical assessment via a medical interview with the parents or a primary caregiver, as well as a direct observation of the child's behaviors within the exam room. There are, however, no available scientific tools for the evaluation of the clinical symptoms in children with ASD, which may lead to an under- or an overestimation of the disease severity.

The well-accepted pathogenesis of autism is the abnormality in neural communication [2]. Impaired long-distance brain connectivity is also commonly observed in a number of autism researches [3–5]. An evaluation of brain connectivity is usually performed by a magnetic resonance imaging (MRI) based technique called diffusion tensor imaging (DTI). However, this neuroimaging technique requires an experienced neuroradiologist to perform and to interpret the results, demanding much time for data analysis, and is relatively quite expensive. In addition, DTI also requires subjects to limit their movements. It is, therefore, a rather difficult technique to perform on children with autism. Moreover, evidence from several researches shows abnormalities not only in structural but also in functional connectivity in children with autism [6].

Normal brain function not only requires an appropriate level of synaptic contacts, but also an appropriate speed of neural transduction. Increased electrical conduction is strongly dependent on the presence of myelin around the nerve fibers. Myelin is a fatty substance derived from the plasma membrane of glial cells and is crucial for rapid neural communication. Myelination, the production of myelin sheath around the axons, is an important brain maturation process which first occurs within the motor and sensory pathways, including the visual pathway, since the early developmental period.

Visual evoked potential (VEP) is an electrophysiological method used in measuring the brain electrical signals recorded over the occipital lobe in response to basic visual stimuli. Generally, VEP is used for the determination of optic nerve lesions, as well as in the evaluation of the visual functions. In clinical practice, VEP is commonly used to reveal the demyelination of optic nerves that is commonly seen in patients with optic neuritis and multiple sclerosis [7]. However, several studies have also revealed VEP abnormalities in various neurological and psychiatric disorders such as Alzheimer's disease [8, 9], migraine headache, and schizophrenia [10–12]. Interestingly, the abnormalities of VEP can also be found in children with developmental delays. A previous study has shown prolonged VEP latencies in slow learners [13]. In children with attention deficit hyperactivity disorder (ADHD), the VEP waveforms are slightly more prolonged than in healthy children [14]. Moreover, alterations in the VEP waveforms were also found in patients with dyslexia [15] and fragile X syndrome [16]. The prolongation of the VEP waveforms usually reflects delayed nerve conduction, which is commonly observed in children with developmental delays.

Children with autism usually show some abnormalities in the visual system such as poor eye control and hypersensitivity to light. These abnormal findings may result either from atypical sensory processing or from defects in neural networks of social cognition. A recent study using a steady-state VEP showed hyperresponsivity to visual stimuli in patients with high-functioning autism [17]. However, there is a current lack of studies that use VEP testing in children with ASD as a brain maturation index. To determine its correlation with potential clinical symptoms of ASD, we therefore planned to investigate neural transmission within the visual system using VEP as an index of the myelination process of the visual pathway and its correlation to the clinical severity of autism. The research hypothesis is that children with ASD show a delayed latency of the VEP waveforms compared to normal children. Moreover, the prolongation of VEP in children with ASD should correlate with some aspects of developmental delay in autism, which can provide a benefit to the VEP in assessing the clinical symptoms of autism.

## 2. Methods

### 2.1. Participants

The participants in this study consisted of children with ASD and typically developed children (controls). The total number of subjects, aged between 3 and 5 years, was 21: 9 children with ASD (all males) and 12 age-matched controls (8 males : 4 females). The diagnosis of ASD in the current work was made during the recruitment period of the participants by a developmental behavioral pediatrician or a child and adolescent psychiatrist in accordance with the DSM-IV-TR, the latest version of DSM at the recruitment period [18].

Children with ASD in this study were initially screened for comorbid conditions. The exclusion criteria for this work included (1) genetic syndromes associated with autistic features such as tuberous sclerosis, (2) autism with neurodegenerative conditions such as Rett's syndrome and childhood disintegrative disorder, (3) patients with other psychiatric disorders, (4) cases with active epilepsy, (5) impaired gross motor function, and (6) the taking of benzodiazepines. Any participant who passed the initial screening process was recruited to receive a complete systemic and neurological examination by a pediatric neurologist (VS). Subjects who had abnormal eye signs such as the presence of nystagmus, significant myopia, and abnormal retinal examination, as well as the presence of focal neurological deficits, were excluded from this study. In addition, handedness was assessed using the Edinburgh Handedness Inventory for the determination of the participants' hand dominance in both groups [19]. Only right-handed children were selected for this study.

All experiments in this study were conducted in accordance with the Declaration of Helsinki and approved by the ethical standards of the Mahidol University Central Institutional Review Board (MU Central-IRB). The research project was given ethical permission number MU-IRB 2013/163.1712. On the date of experimentation, the whole protocol was clearly explained to the parents of all the participants in both groups. They had to sign written consent forms before the experiment could commence. The parents were clearly informed that the experiment could be terminated anytime if their children felt uncomfortable or could not maintain their cooperation.

### 2.2. Evaluation of the Clinical Severity of ASD

In Thailand, the Pervasive Developmental Disorder Screening Questionnaire (PDDSQ) was developed by the Yuwaprasart Waithayopathum Child Psychiatric Hospital of Thailand to be used as a screening tool for children with ASD [20]. It was adapted from several assessment tools for ASD, for example, from the Checklist for Autism in Toddlers (CHAT), the Childhood Autism Rating Scale (CARS), the Autism Screening Questionnaire (ASQ), and the Social Responsiveness Scale (SRS). PDDSQ was established as a checklist for parents or primary caregivers to evaluate the behaviors of their children aged between 1 and 18 years. In this study, all children were screened for history of PDD using this questionnaire.

The clinical severity of autism was evaluated using the Autism Treatment Evaluation Checklist (ATEC). The ATEC was created by Rimland and Edelson in 1999 and was translated into a Thai version with permission in 2004 [21, 22]. The ATEC checklist has 4 subtests: speech/language communication, sociability, sensory/cognitive awareness, and health/physical/behavior domains. The severity scale of ATEC was divided into 3 levels of severity: mild, moderate, and severe. ASD children with severe clinical symptoms were excluded from the current work due to the high chance of poor cooperation. Importantly, regarding the purpose of assessing the clinical severity of autism, ATEC was completed only in the ASD group but not in the control group.

In regard to social development, which is one of the key deficits in children with ASD, the Vineland Adaptive Behavior Scales 2nd edition (VABS-II) was used to assess the adaptive behavior skills of the children [23]. VABS-II is a semistructured interview with a parent, primary caregiver, or teacher. There are 4 domains in VABS-II: communication, daily living skills, socialization, and motor skills. However, only the socialization domain was selected to evaluate the level of social development of all participants. The socialization domain contains three subdomains: interpersonal relationships, play and leisure time, and coping skills. On the contrary to ATEC, children with ASD as well as typically developing children were evaluated by VABS-II for social adaptive behaviors.

### 2.3. Visual Evoked Potential (VEP)

VEP is the electrophysiological method of evaluating the integrity of the visual pathway. However, researches using VEP in patients with brain developmental disorders have also been reported. There are two main types of VEP that are commonly used in clinical studies: flash VEP and pattern-reversal VEP. For the current study, we selected the pattern-reversal VEP because it is more suitable for VEP recording in young children, especially in cases with high degree motor activities and poor inhibitory control. The black and white checkerboard was evenly presented in horizontal grates at a viewing distance of 60 cm from the computer monitor. The checkerboard was presented using the STIM 2.4 software (Neurosoft Inc.). The checkerboard pattern consisted of 8 × 8 checks, including each individual check being subtended at 2.75° visual angle (100% contrast). According to the visual pattern-reversal program, the reversal interval value was set at 500 milliseconds, resulting in a reversal every 0.5 seconds. All participants were prompted to focus on a red-cross fixation point at the center of the display screen during the 3-minute contrast-reversing checkerboard presentation.

*VEP Acquisition.* For VEP recording, a full setup of the electroencephalography (EEG) and evoked potential (EP) was performed. The EEG recording system used in this experiment was the Neuroscan version 4.3 (Neurosoft Inc.). The electrodes were attached onto the Electro-Cap according to the international 10-20 system, with 4 additional electrodes for the electrooculography (EOG) recording [24]. Both mastoid regions were used as reference sites. QuikCell, a cellulose based transmission, was inserted into all 32 channels attached on the Electro-Cap, followed by the use of a blunt needle tip to drop in liquid electrolytes for cell hydration. After the QuikCells were hydrated, they acted as a conducting bridge. The impedance was kept below 10 kΩ. The prerecording band-pass filter was set from 0.1 to 60 Hz. A notch was opened at 50 Hz. The EEG and the EOG were recorded at a sampling rate of 1,024 Hz.

*Analysis of the VEP Waveforms.* In regard to the analysis of the VEP waveforms, the main electrodes used for the analysis consisted of the Oz, O1, and O2 electrodes, representing the midline, left, and right occipital regions, respectively. Artifact rejection was set to reject the epoch with the channel amplitude exceeding ±80 microvolts. The EEG segments time-locked to the onset of visual stimuli were selected from 100 milliseconds (ms) before and 300 ms after the stimulus onset. At least 80 epochs of artifact-free EEG data from each subject were allowed for further steps of VEP analysis. The period of EEG segment prior to the stimulus onset was used as baseline and the postrecording band-pass filter was assigned from 0.3 to 30 Hz. VEP data was analyzed in the time domain. The amplitude and latency of the VEP waveforms were used as a basis of comparison between groups. Amplitude (microvolt) is defined as the difference between the height of the current peak and the highest point of the previous peak, while latency (millisecond) is defined as the duration from the visual stimulus to the peak of each VEP waveform. In general, the VEP waveforms generated in the pattern-reversal VEP consist of N75, P100, and N145 waves.

### 2.4. Statistical Analysis

All data generated from this study was statistically proven for normal distribution using the Kolmogorov-Smirnov test. The comparisons of the VEP waveforms were analyzed using independent-samples* t*-test between groups (ASD versus control). In addition, Pearson's correlation was used to assess the correlation within the ASD group between the VEP parameters and the ATEC scores, as well as the correlation between the VEP parameters and the behavioral score of VABS-II in social performance for all participants.

## 3. Results

The age distribution in both groups was not statistically different by means of the Kolmogorov-Smirnov test. There was also no significant difference in terms of the handedness and the socioeconomic profiles. As expected, the scores of PDDSQ and VABS-II, especially in the socialization domain, were significantly different (*p* < 0.05) between the two groups. Individuals with ASD had higher PDDSQ scores than controls, which is consistent with the prediction along the lines of the autistic trend. In addition, the ASD group showed lower scores than controls on the VABS-II subtest in the socialization domain, which explains their below-average social behaviors compared to children in their age group. The clinical characteristics of the participants including age, gender, handedness, and behavior screening scores are displayed in Table 1.

**Figure 1:**
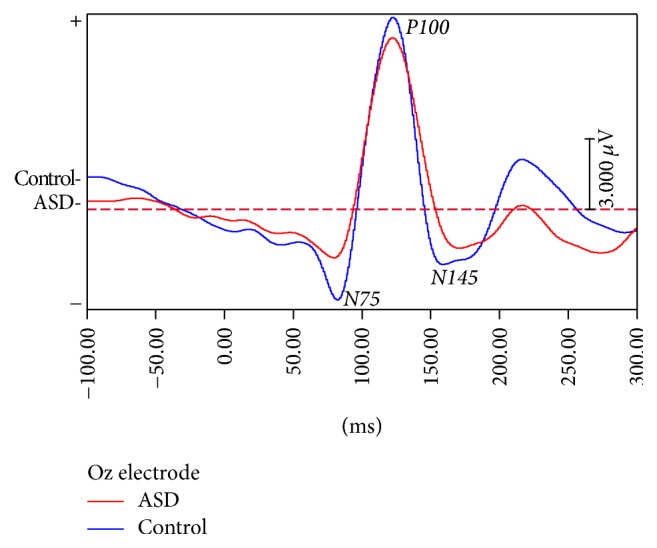
The VEP waveforms over the Oz electrode, a comparison between the ASD and the control groups.

For the VEP analysis, the amplitude and the latency of the VEP waveforms were used as parameters in comparing the outcomes between the groups. At the site of the Oz electrode, our results showed that the latency of the N145 wave was significantly prolonged in the autism group, compared to the control group (df = 19, *p* = 0.014). In addition, the latency of the P100 wave was slightly prolonged in the autism group but did not reach the significant level (df = 19, *p* = 0.08). Regarding the amplitude, the amplitude of the N75-P100 wave in the autism group slightly reduced (df = 19, *p* = 0.51), compared to the controls. All the VEP parameters are displayed in Table 2 and the VEP waveforms generated at the site of the Oz electrode are shown in Figure 1.

Apart from the Oz electrode, the latency of the N145 wave recorded over the O2 electrode in the autism group was longer than in the control group (df = 19, *p* = 0.02). Other parameters did not reveal a significant difference between the two groups. The VEP waveforms generated at the O1 and O2 electrodes are shown in Table 3.

**Figure 2:**
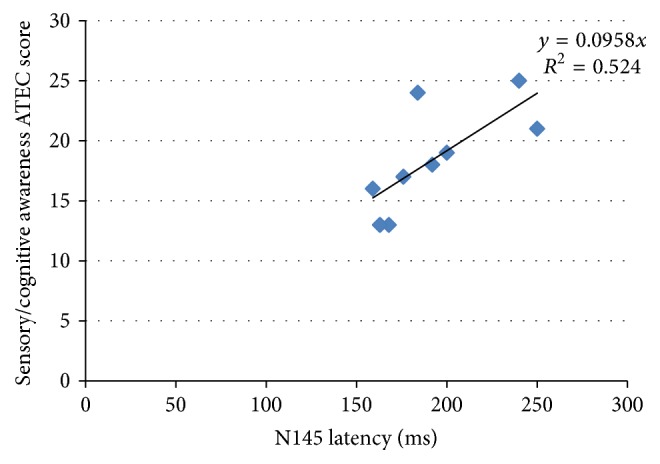
Scatter plot displaying the correlation between N145 latency over Oz electrode and the ATEC scores for subtests in sensory/cognitive awareness.

The relationships between the VEP parameters and the ATEC scores were evaluated using Pearson's correlation only in the ASD group (Table 4). There was a positive significant correlation between N145 latency and ATEC score. Longer N145 latency was associated with a higher score within the sensory/cognitive awareness subdomain as measured using ATEC assessment (*r* = 0.724, *p* = 0.03) as shown in Figure 2. These findings indicate that more severe autistic symptoms are correlated with a delayed VEP response in the N145 wave over the Oz electrode. However, no such correlation was observed between other VEP waveforms (N75 and P100 waves) and other subdomains of ATEC.

**Figure 3:**
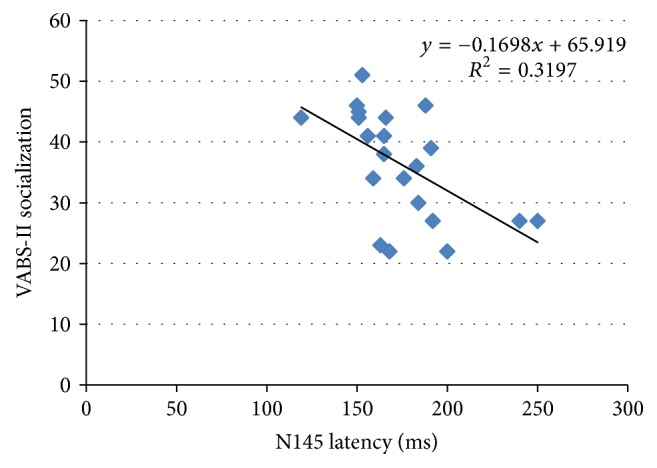
Scatter plot displaying the correlation between N145 latency over Oz electrode and VABS-II standard scores in the socialization domain.

Across the entire body of participants, the latencies of the P100 and the N145 waves over the Oz electrode were found to correlate with the standard scores measured using VABS-II. In this study, the standard scores of VABS-II were focused on the socialization domain. On the interpretive scores, the lower socialization domain standard scores of VABS-II were described as more delayed in social interaction performance as well as more severe symptoms of social deficits. The higher adaptive behavior scores in the socialization domain reflected the better developmentally appropriate social functioning of children. There was a negative significant correlation between the P100 latency and the VABS-II scores only in the subdomain of socialization. Specifically, the longer P100 latency correlated with a lower score within the interpersonal relationships subdomain (*r* = −0.436, *p* = 0.048), but not in other subdomains. Interestingly, the correlation between N145 latency and VABS-II scores was negatively significant for the socialization domain (*r* = −0.565, *p* = 0.008) and all its subdomains, consisting of interpersonal relationships (*r* = −0.539, *p* = 0.012), play and leisure time (*r* = −0.565, *p* = 0.008), and coping skills (*r* = −0.476, *p* = 0.03) as shown in Table 5 and Figure 3.

## 4. Discussion

In this study, we investigate the neural transmission within the visual system, measured using pattern-reversal VEP in children with ASD, compared to age-matched controls. VEP is a method used in studying the function of the visual pathway and can be performed in poorly cooperative participants like children with ASD. The amplitude and the latency of VEP waveforms are used as the main parameters for comparison. The latency of the VEP waveform indicates the velocity of the neural transmission in the optic nerve, a part of the visual pathway. Previous research showed that prolonged latency of the VEP waveforms is commonly found in patients with demyelination of the optic nerve, including patients with optic neuritis and multiple sclerosis [25, 26]. Thus, myelination is the most important factor for determining the latency of the VEP waveforms. However, the physiological factors including age, gender, visual acuity, and medications as well as the cognitive factors have been reported to affect the VEP latency [27, 28]. In this study, we examined the neural communication within the visual pathway to evaluate the level of myelination of the visual circuit, and we tried to determine the possible correlations between the VEP parameters and the ASD symptoms for the possibility of using VEP as a method in the clinical evaluation of ASD severity.

Myelination is a process of brain maturation, which occurs early in life and continues to develop until early adulthood [29, 30]. The pattern of myelination in the brain occurs in a region-specific manner [30]. In general, myelination begins in the sensory pathways, which include the visual system, followed by the motor pathways. The presence of myelin around the neuronal axons promotes the speed of neural transmission. In the visual pathway, myelination usually develops since early life, similar to the neural circuits in other sensory domains. Thus, if the visual function is normal, VEP can be used for the evaluation of the degree of myelination in early childhood, which corresponds with the maturation of neural communication.

A previous study using VEP in adults with ASD revealed an alteration in the VEP response, compared to controls. More specifically, a visual event-related potential (ERP) study in adults with Asperger's syndrome showed an alteration of early and late visual processing shown by the reduced amplitude of ERP waves [31]. Similarly, our study demonstrated the occurrence of different VEP signals in children with ASD, compared to typically developing (TD) children. The latency of N145 wave in children with autism was significantly longer over the Oz and O2 electrodes compared to age-matched controls. The prolonged N145 latency indicates delayed neural processing in the visual pathway. Some children with ASD even revealed some abnormality in the eyes and optic pathways that might affect their VEP results. Our study tried to reduce this confounding factor by excluding any participants who had abnormal eye signs. This elimination approach can help us confirm that delayed N145 latency is due to the slower neural communication in the visual circuit, but not because of any ocular or optic pathway lesions, which can be found in children with ASD.

Interestingly, the prolongation of VEP latency found in this current work correlates with higher ATEC scores within the sensory and cognitive awareness domains. The ATEC score is the measurement of the clinical severity of autism; therefore, this finding could be considered in linking clinical symptoms of autism with the electrophysiological parameters. It appears in this study that the N145 latency was slower in children with autism than in the TD children, which supports the delay of neural communication along the visual pathway. The correlation between delayed neural processing in visual pathway as shown by prolonged N145 latency and higher level of autism severity through ATEC scoring may indicate a similar brain maturation pattern between the visual pathway and the neural circuits within other sensory domains and cognition. This evidence supports our hypotheses in relation to the delayed neural transmission of the visual pathway in ASD children and the possibility of using VEP in evaluating the clinical severity of autism.

Moreover, the VEP response to checkerboards in children with ASD also showed that slower P100 and N145 latency over the Oz electrode correlated with lower VABS-II scores within the socialization domain. This finding indicates the correlation between longer VEP latencies and more delays in social interaction performance as shown by lower VABS-II scores. Although there are a few studies that show correlations between the VEP electrophysiology recordings and the social behavior measurement in autism, the studies using ERP response to human face as the index of the N170 wave have been found to be consistent with social performance in children with autism. Among twins with and without autism, the speed of face processing as indicated by the latency of P1 and N170 waves was found to correlate with social skills. Faster P1 latency is associated with better social participation and greater response of the N170 wave to upright face images, which is related to fewer social difficulties [32].

Some studies have reported the alteration of amplitude of the VEP waveforms in patients with ASD, compared to children with normal developmental milestones. For example, a study using the novel transient visual evoked potential revealed that children with ASD showed smaller VEP amplitude than controls, while there was no group difference between the VEP latencies using this technique [33]. However, our findings did not demonstrate any significant alterations in the VEP amplitude between children with ASD and TD, with the VEP latency being the only parameter with a significant difference. The reason may be explained by the observation that the visual function in both groups of participants was normal, so the amplitudes of the VEP waveforms representing the neural activities within the visual pathway were not significantly different between the groups. The main difference is the speed of neural transmission as revealed by the longer VEP latency in children with ASD. Moreover, the correlations between the VEP amplitude and the severity of the ATEC scores, including social adaptive behavior, were also not observed in this study.

There are some limitations to this study. First, the sample size in this study is rather small. Second, the gender distribution is different between the two groups. There are only males in the ASD group, whereas children in the control group consisted of both genders. Previous studies have demonstrated different VEP waveforms between males and females for both children and adults [27, 34, 35]; however, the unequal gender distribution observed in our work may affect the VEP results. Third, the cognitive factors, especially for attention, have been found to affect the latency of EP as well as ERP waveforms [28]. Thus, a formal assessment with neuropsychological tests should be performed in a further study. Finally, even with careful data analysis, the movement and muscle artifacts may have still persisted in the cases of poorly cooperative young children, particularly with the ASD participants.

## 5. Conclusion

Using VEP as the index of neural processing within the visual system of children with ASD could become a possible tool for the clinical assessment of the disease severity. The prolongation of N145 latency indicates delayed neural communication within the visual pathway. The correlation between VEP latency and the ASD clinical symptoms assessed using the ATEC and the VABS-II scores in the socialization domain may indicate a close association between myelination within the visual circuits and the symptoms of autism. More research evidence is, however, required to confirm the utility of VEP in clinical settings. Further research would also shed light on the technique of electrophysiological evaluation of the ASD symptoms, other than the routine clinical assessments.

## Figures and Tables

**Table 1:** The clinical characteristics of children in both the ASD and the control groups. Data is shown as mean and standard deviation (SD).

Clinical parameters	ASD group (*n* = 9)	Control group (*n* = 12)
Age (months)	47.44 (9.98)	45.5 (8.44)
Gender ratio (male : female)	9 : 0	8 : 4
Handedness (%)	70.72 (37.46)	84.07 (20.12)
VABS socialization (total)	27.33 (4.64)^*∗*^	42.92 (4.12)
PDDSQ (total)	20 (6.98)^*∗*^	4.42 (3.58)
ATEC (total) (percentile)	66.44 (15.72) (50–59)	-

^*∗*^ *p* < 0.05.

**Table 2:** The amplitude and latency of the VEP waveforms over the Oz electrode between the two groups.

VEP waveform (Oz electrode)	Autism group (*n* = 9)	Control group (*n* = 12)
N75 latency	79.11 (8.77)	79.91 (12.43)
P100 latency	131.89 (25.19)	115.75 (14.85)
N145 latency	192.44 (32.7)^*∗*^	161.5 (19.87)
N75-P100 amplitude	10.97 (5.59)	13.24 (8.73)
P100-N145 amplitude	12.65 (6.92)	13.44 (6.83)

^*∗*^ *p* < 0.05.

**Table 3:** A comparison between the VEP waveforms generated in each hemisphere, at the O1 electrode site for left occipital and the O2 electrode site for right occipital areas.

VEP waveforms	Electrode	Autism group	Control group
N75 latency	O1	82.56 (13.44)	81 (21.24)
	O2	81 (10.11)	80.72 (17.53)
P100 latency	O1	127.44 (12.72)	112.75 (21.11)
	O2	138.78 (35.16)	120.83 (15.56)
N145 latency	O1	179.44 (26.13)	163.83 (16.28)
	O2	192.63 (35.77)^*∗*^	161.58 (19.76)
N75-P100 amplitude	O1	5.59 (1.67)	5.88 (3.27)
	O2	9.75 (5.96)	10.37 (5.89)
P100-N145 amplitude	O1	7.28 (3.11)	7.36 (3.58)
	O2	11.27 (8.06)	11.15 (5.16)

^*∗*^ *p* < 0.05.

**Table 4:** A correlation analysis between the VEP parameters over the Oz electrode and the ATEC scores.

	Correlation coefficients “*r*”				
	Speech/language/communication ATEC score	Sociability ATEC score	Sensory/cognitive awareness ATEC score	Health/physical behavior ATEC score	Total ATEC score
N75 latency	0.107	0.563	−0.412	0.171	0.269
P100 latency	−0.258	−0.086	0.439	0.277	0.244
N145 latency	−0.111	−0.383	0.724^*∗*^	0.072	0.082

*Note.* ^*∗*^ *p* < 0.05.

**Table 5:** A correlation analysis between the VEP parameters over the Oz electrode and the VABS-II standard scores in the socialization domain and its subdomains.

	Correlation coefficients “*r*”			
	Interpersonal relationships subdomain	Play and leisure time subdomain	Coping skills subdomain	Socialization domain
N75 latency	−0.001	0.015	0.022	0.007
P100 latency	−0.436^*∗*^	−0.319	−0.321	−0.392
N145 latency	−0.539^*∗*^	−0.565^*∗∗*^	−0.476^*∗*^	−0.565^*∗∗*^

*Note.* ^*∗*^ *p* < 0.05; ^*∗∗*^*p* < 0.01.
